# Lifestyles, Left Atrial Structure and Function, and Cognitive Decline in Adults with Metabolic Syndrome

**DOI:** 10.3390/jcm12186066

**Published:** 2023-09-20

**Authors:** Ines Gonzalez Casanova, Ángel M. Alonso-Gómez, Dora Romaguera, Estefanía Toledo, Linzi Li, Elena Fortuny, Luis López, Raúl Ramallal, Jordi Salas-Salvadó, Lucas Tojal-Sierra, Olga Castañer, Alvaro Alonso

**Affiliations:** 1 Department of Applied Health Science, School of Public Health, Indiana University Bloomington, Bloomington, IN 47404, USA; 2 Bioaraba Health Research Institute, Osakidetza Basque Health Service, Araba University Hospital, University of the Basque Country UPV/EHU, 01009 Vitoria-Gasteiz, Spain; 3 CIBER Consortium, M.P. Physiopathology of Obesity and Nutrition (CIBERObn), Carlos III Health Institute (ISCIII), 28029 Madrid, Spainetoledo@unav.es (E.T.); jordi.salas@urv.cat (J.S.-S.); 4 Health Research Institute of the Balearic Islands (IdISBa), 07120 Palma de Mallorca, Spain; 5 Department of Preventive Medicine and Public Health, University of Navarra, 31008 Pamplona, Spain; raulramallal@hotmail.com; 6 IdiSNA, Navarra Institute for Health Research, 31008 Pamplona, Spain; 7 Department of Epidemiology, Rollins School of Public Health, Emory University, Atlanta, GA 30322, USAalvaro.alonso@emory.edu (A.A.); 8 Cardiology Service, Manacor Hospital, 07500 Palma de Mallorca, Spain; 9 Cardiology Service, Son Espases University Hospital, 07120 Palma de Mallorca, Spain; luislopez@hmanacor.org; 10 Department of Cardiology, University Hospital of Navarra, Servicio Navarro de Salud Osasunbidea, 31008 Pamplona, Spain; 11 Group ANUT-DSM, Human Nutrition Unit, Department of Biochemistry and Biotechnology, Universitat Rovira i Virgili, 43201 Reus, Spain; 12 Human Nutrition Unit, Institut d’Investigació Sanitària Pere Virgili (IISPV), 43204 Reus, Spain; 13 Cardiovascular Risk and Nutrition Research Group, Institut Hospital del Mar d’Investigacions Mèdiques (IMIM), 08003 Barcelona, Spain; ocastaner@imim.es; 14 CIBER Consortium, M.P. Epidemiology and Public Health (CIBEROESP), Carlos III Health Institute (ISCIII), 28029 Madrid, Spain

**Keywords:** lifestyle, diet, physical activity, weight, cognitive decline, left atrium

## Abstract

Evidence supports associations of lifestyle (including diet and physical activity) and weight with cognitive functioning, but the pathways responsible for these associations have not been fully elucidated. Because healthier lifestyles have been associated with better left atrial structure and function, which in turn is associated with better cognitive functioning, we tested the hypothesis that left atrial structure and function is a potential mediator of the association between lifestyle and cognition. We included 476 participants classed as overweight or obese with metabolic syndrome from three centers in Spain. These participants underwent lifestyle assessments and transthoracic echocardiography at baseline and repeated measurements of the Trail Making A test, a measure of executive function, taken at baseline and at the two-year follow-up. We conducted mediation analyses to test if measures of left atrial structure and function mediated associations between adherence to the Mediterranean diet scores, physical activity, and weight at baseline, as well as a two-year change in Trail Making A scores. The analysis did not find an association between these factors and Trail Making A scores, and no indirect effects appeared to be mediated by echocardiographic measurements. The modest sample size in this analysis is a limitation, and larger studies should be conducted to determine potential cardiovascular factors mediating the association between lifestyle and cognition.

## 1. Introduction

Cognitive impairment, defined as having difficulties with memory, learning, or cognitive tasks beyond those expected based on age and educational level [1], has become a primary health concern for aging populations. It is estimated that approximately 11% of US adults over 65 years suffer from dementia and 23% have mild cognitive impairment [2]. Elements of a healthy lifestyle, including healthy eating, maintaining weight, and engaging in physical activity, have been associated with reduced cognitive decline as well as dementia prevention, primarily in observational studies [3]. In terms of healthy eating, the strongest associations have been shown with adherence to the Mediterranean diet or Mediterranean-style dietary patterns like the MIND diet [4]. Two meta-analyses of observational studies concluded that adherence to the Mediterranean diet was associated with the prevention of cognitive impairment, dementia, and Alzheimer’s disease [5,6]. Similarly, obesity and physical inactivity are two out of twelve modifiable risk factors that account for 40% of worldwide dementias [7]. Being overweight has been associated with a higher risk of mild cognitive decline and dementia [8], and regular moderate physical activity is associated with better cognitive function in adults older than 60 years [9]. In fact, physical inactivity was identified as the factor with the highest population attributable risk for Alzheimer’s disease in the US, Europe, and the United Kingdom [10].

Healthy eating, weight, and physical activity are also associated with healthier left atrial (LA) structure and function [11]. A recent review evaluated the role of different nutrients as modulators of cardiac remodeling and concluded that antioxidant dietary compounds, such as polyunsaturated fatty acids, vitamin a, folic acid, magnesium, selenium, and zinc, and some dietary patterns, such as ketogenic and low-calorie diets, were associated with lower cardiac remodeling, less oxidative stress, and better function in animal models [12]. Similarly, adherence to the Mediterranean diet was inversely associated with atrial fibrillation (AF) among low-risk participants in a matched case-controlled study [13]. The association between physical activity and atrial remodeling is more complex. Exercise can induce healthy physiological remodeling and cardiac enlargement, while physical inactivity is associated with pathological atrial enlargement among people at high or moderate risk for cardiovascular disease [14,15,16]. Meanwhile, obesity has been identified as an independent risk factor for AF because of its negative impact on cardiovascular hemodynamics and structure [17]. In fact, the Atherosclerosis Risk in Communities (ARIC) study found that approximately one in five cases of AF could be attributable to overweight or obesity [18].

**Figure 1:**
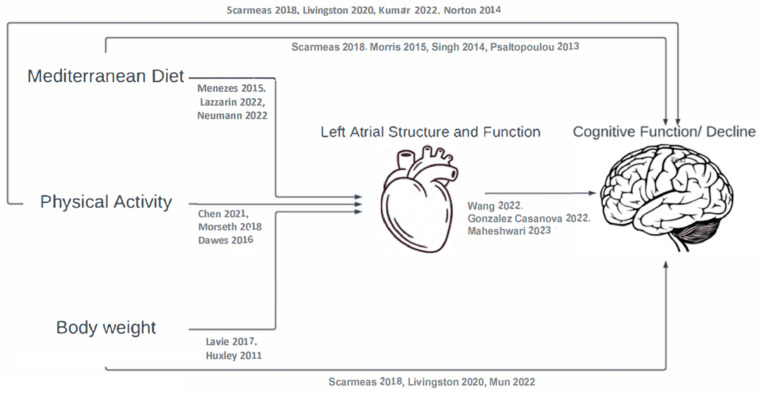
Left atrial structure and function as a potential mediator of the association between lifestyle and cognitive decline [3,4,5,6,7,8,9,10,11,12,13,14,15,16,17,18,19,20,21].

In turn, markers of atrial remodeling and fibrosis, such as enlarged LA size and volume and abnormal LA function, have been associated with cognitive decline and dementia [19]. In a recent analysis among a subsample of participants of the PREDIMED Plus trial, we found that larger left atrial volume index, lower peak longitudinal strain, and higher stiffness index were associated with 2-year worsening performance in the Trail-Making Test A, a measure of executive function [20]. In summary, as illustrated in Figure 1, there is evidence supporting associations between lifestyle and cognitive functioning, as well as evidence of the potential role of left atrial structure and functioning mediating these associations [19,21]. Hence, the objective of this analysis was to test the hypothesis that the associations of weight and other lifestyle factors with 2-year cognitive decline were mediated by measurements of LA structure and functioning in this same sample of participants with overweight or obesity.

## 2. Materials and Methods

### 2.1. Study Design, Setting, and Participants

This was a secondary data analysis of the PREDIMED Plus trial, an ongoing multicenter randomized controlled trial aimed at preventing cardiovascular disease in overweight or obese adults with metabolic syndrome (ISRCTN89898870). PREDIMED Plus is a randomized clinical trial in which 6874 participants were recruited in 23 centers and hospitals across Spain between September 2013 and November 2016, and randomized to an intensive lifestyle intervention seeking weight loss based on either an energy-restricted Mediterranean diet supplemented with extra-virgin olive oil and nuts, together with promotion of physical activity and a behavioral intervention (intervention group), or a Mediterranean diet supplemented with extra-virgin olive oil and nuts without calorie restriction or physical activity (control group) [22,23]. Eligibility criteria for the main trial broadly included men (aged 55–75 years) and women (aged 60–75 years; the reason behind this age cut-off difference is because women younger than 60 have very low risk of CVD) without documented history of cardiovascular disease at enrollment, who were overweight/obese (BMI ≥27 and <40 kg/m^2^) and disclosed at least three symptoms of metabolic syndrome according to its harmonized definition in the joint statement from the International Diabetes Federation/National Heart, Lung, and Blood Institute/American Heart Association (2009) [22,23]. Exclusion criteria included refusal to provide written informed consent, illiteracy, documented history of CVD (angina, myocardial infarction, coronary revascularization procedures, stroke (ischemic or hemorrhagic, including transient ischemic attacks), symptomatic peripheral artery disease that required surgery or was diagnosed with vascular imaging techniques, ventricular arrhythmia, uncontrolled atrial fibrillation, congestive heart failure (New York Heart Association Class III or IV), hypertrophic cardiomyopathy, or a history of aortic aneurysm ≥ 5.5 cm in diameter or aortic aneurism surgery), active malignant cancer, inclusion in another weight loss program, and food allergies relating to the Mediterranean diet, among others [22,23]. Hypertension was not an exclusion criterion. For this analysis, we included a sub-sample of 476 participants from three recruiting centers (the University of Navarra, Araba University Hospital, and Son Espases University Hospital) who underwent transthoracic echocardiography at baseline and who had also taken the Trail Making A test at baseline and at the two-year follow-up.

A priori power calculations were conducted as part of a broader study aimed at testing the effect of a lifestyle intervention on echocardiographic measurements of left atrial structure and function. Specifically, minimum detectable size calculations were based on prior results from the PREDIMED Plus trial and from an intensive lifestyle intervention trial in patients with atrial fibrillation [24,25], and sample size was calculated using formulas provided by Fitzmaurice, Laird, and Ware [26]. Calculations were based on a 1:1 parallel design, with repeated measures at years 0, 3, and 5, power = 0.9, 2-tailed Ro = 0.005 (to account for the multiple echocardiographic variables and biomarkers considered), a within-person correlation (Ro) of 0.1 in the outcome variables, and a conservative sample size of 430 (25% attrition among 573 participants with baseline echocardiographic studies). Based on these assumptions, we expected to be able to detect between-group differences in the slope of the annual rate of change of 0.105 standard deviations.

### 2.2. Data Sources and Measurements

Trail Making Tests measure processing speed and executive function [27]. Trail Making Test A, where participants are asked to connect numbers 1 to 25 in the correct order, is meant to assess cognitive processing skills. The score for this test is obtained based on the number of seconds that it takes to complete the test. Scores were multiplied by −1 for higher scores to represent better performance. Two-year follow up scores were standardized by subtracting the baseline means and dividing them by the baseline standard deviation. To calculate 2-year cognitive change, standardized baseline scores were deducted from 2-year follow-up scores ([(2y score − baseline X)/baseline SD] − [(baseline score − baseline X)/baseline SD]).

Echocardiographic examinations were performed by practicing cardiologists at baseline using an ultrasound scanner Vivid 7 or Vivid 9 (General Electric Healthcare) following common procedures extensively described elsewhere [28]. Briefly, M-mode, doppler imaging, and two-dimensional cine loops for three heart beats of standard views were obtained from each patient. All images were digitally stored and offline ultrasound software EchoPac (GE Healthcare v.204) was used for analysis. Images were read at a core reading center (Son Espases University Hospital) by two trained readers (also practicing cardiologists) who were blinded to clinical data and followed the recommendations of the American Society of Echocardiography [29].

The LA volume index—defined as LA volume indexed to body surface area—was included as a marker of LA structure. Peak LA longitudinal strain, conduit strain, contractile strain, the LA function index, and the LA stiffness index were included as measures of LA function. The peak LA longitudinal strain, a marker of LA myocardial function, was measured at the end of the reservoir phase. Early diastole strain represents conduit function and late diastole strain represents contractile function. LA stiffness index, a measure of LA remodeling and fibrosis, was calculated as E/e’ ratio divided by peak LA systolic longitudinal strain, where E represents the early mitral inflow velocity (E wave) and e’ represents the medial and lateral mitral annular velocity [30].

Adherence to the energy-reduced Mediterranean diet was assessed through a 17-item short screener, where higher scores indicated better adherence [31]. Metabolic equivalents (METs) per day of moderate to vigorous physical activity were estimated using the short version of the Minnesota leisure time physical activity questionnaire [32]. Height and weight were measured during the baseline visit. Participants’ sociodemographic and health characteristics were assessed at baseline via a face-to-face questionnaire and included age, sex, research site, number of people living in the household, employment, years of schooling, smoking (currently or historically), self-reported diagnoses of diabetes, depression, and presence of arrythmias other than AF (because AF was an exclusion criterion for the intervention). The intervention group was also recorded and included in the analysis.

### 2.3. Quantitative Variables and Statistical Methods

We conducted mediation analyses using the methodology and SAS (v.9.4, SAS Institute Inc., Hong Kong, China) macro developed by Valeri and Vanderweele (2013) [33]. Three different models were utilized to estimate the total effects of adherence to the Mediterranean diet score, MVPA metabolic equivalents per day, and body mass index on Trail Making Test A, as well as to decompose the effects into direct effects and indirect effects mediated through echocardiographic variables that have been shown to be associated with change in cognitive scores in our previous analysis (left atrial volume index, peak systolic longitudinal strain, conduit strain, contractile strain, and stiffness index) [20]. Exposure variables were converted into z-scores through subtracting the mean from individual values and dividing the result by the standard deviation. Hence, model estimates represent the two-year difference in Trail Making Test A standard deviations per standard deviation change on the exposure variable. Models were adjusted for age, sex, site, number of people living in the household, employment status (yes or no), education (primary, high school, professional), smoking status (never, former, current), diagnosis of arrhythmia, self-reported diagnosis of diabetes, self-reported diagnosis of depression at baseline, and intervention group.

## 3. Results

Participants were 61% male, had an average age of 65 years, and had an average of 12 years of schooling (Table 1). Most participants had hypertension and about a fifth had diabetes or depression. A small percentage reported arrythmias other than AF, which were not documented as CVD at baseline. The average change in Trail Making A score, which is measured in seconds required to complete the task, was 1.9 (SD = 20.9).

The Trail Making Test A scores were 48.1 (21.2) at baseline and 49.9 (23.9) at the 2-year follow-up visit (difference: 1.8, 95% CI 0.0, 3.7). We did not find evidence for an effect of adherence to the Mediterranean diet, physical activity, or body mass index on 2-year change in the Trail Making Test A score (Table 2, Total effect column). Similarly, using effect decomposition, we did not find any significant direct effect or echocardiography-mediated indirect effect of adherence to the Mediterranean diet, physical activity, or body mass index on the outcome (Table 2).

Estimates represent the change in outcome per a standard deviation difference in exposure adjusted by age, sex, site, number of people living in the household, employment status (yes or no), years of schooling, current smoking (yes or no) and former smoking (yes or no), diagnosis of arrhythmia, self-reported diagnosis of diabetes, self-reported diagnosis of depression at baseline, and intervention group. Models were also adjusted for METS per day of moderate to vigorous physical activity, Mediterranean diet 17-item score (higher scores indicate better adherence), and body mass index, except when these were the exposure variables.

## 4. Discussion

Based on previous evidence of the association between diet, physical activity, and body mass index with cognitive decline, and on our previous findings demonstrating associations between LA markers and cognitive decline in this cohort [20], we tested the hypothesis that LA markers mediated the association between these lifestyle factors and cognitive decline. Results from this analysis among 476 persons at high cardiovascular risk showed no association of adherence to the Mediterranean diet, daily moderate to vigorous physical activity, or body mass index with 2-year change in Trail Making Test A. Even though we had previously observed an association between the LA markers included in this analysis and 2-year changes in the Trail Making Test A, we found no significant indirect association using LA measurements [20]. This was most likely a consequence of the null overall association with lifestyle.

This result was unexpected, especially for adherence to the Mediterranean diet, which has been shown in several studies to be associated with cognitive function [5,6]. A systematic review and meta-analysis in 2014 that included five studies found that adherence to the Mediterranean diet was inversely associated with cognitive decline, where subjects with the highest Mediterranean diet adherence had a 27% lower risk of mild cognitive impairment [5]. An earlier cohort study also found that those with the highest adherence to the Mediterranean diet had less cognitive decline after 5 years compared to those with the lowest adherence [34]. A more recent cohort study that included 6321 Hispanics in the US found that high adherence to the Mediterranean diet was associated with better global cognition and decreased 7-year memory decline compared with those with low adherence [35].

Physical activity has also been identified as beneficial for cognitive functioning across the life cycle [36], with observational studies showing that greater amounts of daily physical activity are associated with lower risks of cognitive impairment [37]. However, questions remain about how these benefits vary depending on age, exercise type, and intensity [36]. In this case, we only had a measurement of weekly self-reported physical activity, which may explain the null association with 2-year change on a test for processing speed and executive function.

Additional potential explanations for the null associations are the limited sample size (although it was larger than originally expected and estimates are consistent with null associations), a short follow-up of only two years leading to imprecise estimates of effect, and associations tested in this analysis being specific to 2-year change in a measure of executive function, while previous studies have looked at overall scores, having mostly used the Mini-mental Examination that measures overall cognitive functioning [5,6]. Another difference compared with previous studies was that this analysis was limited to older adults with metabolic syndrome, which may have decreased the variability in our sample, although there is still wide variability in lifestyle and body mass index between people with metabolic syndrome. Moreover, older adults with metabolic syndrome are at high risk of cardiac remodeling and minor cognitive impairment.

Strengths of this analysis include the good retention of study participants, repeated measurements of cognition, information available on several sociodemographic and health covariates, and the use of echocardiography to assess LA structure and function. Potential limitations include the short follow-up time of only 2 years, the lack of precision in the estimates due to the modest sample size, and the exclusion of participants with cardiovascular disease or low cognitive functioning based on the design of the trial.

## 5. Conclusions

Even though evidence supports associations between lifestyle and cognitive decline, we did not find them in this study, conducted among older adults with metabolic syndrome, a sample that is at high risk for cognitive decline. Similarly, there was no evidence of mediation by markers of left atrial structure and function. Other analyses that are based on randomized designs and include longer follow-ups, larger samples, and diverse measurements of cognitive functioning are needed to elucidate the potential mediating role of LA measurements in the associations between lifestyle and cognitive functioning.

## Figures and Tables

**Table 1:** The sociodemographic and health characteristics of participants included in the analysis of echocardiographic markers of left atrial structure and change in Trail Making test A scores (*n* = 476).

% or Mean (SD)	Total (476)
Site	
Mallorca	27.1
Navarra	20.8
Vitoria	52.1
Age (years)	65.2 (4.9)
Married	77.9
People living in the household	1.3 (1.0)
Schooling (years)	12.0 (5.2)
Employed	17.7
Health status	
Body Mass Index (kg/m^2^)	32.5 (3.3)
Diabetes (self-reported)	22.7
Hypertension (self-reported)	83.6
Depression (self-reported)	17.9
Arrythmia (self-reported)	5.5
Health Behaviors	
Current Smoker	9.7
Former Smoker	51.3
Mediterranean Diet Score (17-item screener)	7.5 (2.9)
Moderate to vigorous physical activity (MET-min/day)	269 (318)
General Echocardiographic Measures of Left Atrial Substrate	
Volume Index (mL/m^2^)	23.3 (7.5)
Peak Systolic Longitudinal Strain (%)	27.3 (6.8)
Conduit Strain (%)	−11.9 (4.4)
Contractile Strain (%)	−15.4 (4.9)
Stiffness Index (U)	0.4 (0.2)
Trail Making Test A (seconds)	
Baseline	48.1 (21.2)
2-year difference	1.9 (20.9)

**Table 2:** Analysis of left atrial volume and strain lifestyle variables as mediators in the association between body mass index, Mediterranean diet score, METs in moderate to vigorous physical activity/day, and Trail Making Test 2-year change.

	Mediterranean Score			MVPA MET—Min/day			Body Mass Index		
Effect Estimate (95% CI)	Direct	Indirect	Total	Direct	Indirect	Total	Direct	Indirect	Total
Volume Index (mL/m^2^)	0.01 (−0.10, 0.13)	−0.01 (−0.03, 0.00)	0.00 (−0.11, 0.11)	−0.05 (−0.16, 0.05)	−0.02 (−0.03, 0.00)	−0.07 (−0.17, 0.03)	0.01 (−0.08, 0.09)	0.00 (−0.01, 0.01)	0.00 (−0.09, 0.09)
Peak Systolic Longitudinal Strain (%)	−0.02 (−0.13, 0.08)	0.01 (−0.01, 0.02)	−0.01 (−0.12, 0.09)	−0.05 (−0.15, 0.05)	0.00 (−0.01, 0.01)	−0.05 (−0.15, 0.05)	0.00 (−0.08, 0.09)	−0.01 (−0.02, 0.00)	0.01 (−0.09, 0.08)
Conduit Strain (%)	0.01 (−0.12, 0.09)	0.01 (−0.01, 0.01)	−0.01 (−0.12, 0.10)	−0.05 (−0.15, 0.05)	0.00 (−0.01, 0.01)	−0.05 (−0.15, 0.05)	0.00 (−0.08, 0.08)	0.00 (−0.01, 0.00)	0.00 (−0.09, 0.08)
Contractile Strain (%)	−0.01 (−0.12, 0.10)	0.00 (−0.01, 0.01)	−0.01 (−0.11, 0.10)	−0.05 (−0.15, 0.05)	0.00 (−0.01, 0.01)	−0.05 (−0.15, 0.05)	0.00 (−0.08, 0.09)	−0.01 (−0.02, 0.00)	−0.01 (−0.09, 0.08)
Stiffness Index (U)	−0.02 (−0.12, 0.09)	0.00 (−0.02, 0.02)	−0.02 (−0.12, 0.09)	−0.06 (−0.15, 0.04)	0.01 (−0.01, 0.02)	−0.05 (0.15, 0.05)	0.00 (−0.08, 0.09)	−0.02 (−0.03, 0.00)	−0.01 (−0.09, 0.07)

## Data Availability

This is a secondary analysis of the PREDIMED Plus trial, which is ongoing. Data will be available as described on the Data Management Plan and Data Sharing Policy (link below): https://www.predimedplus.com/wp-content/uploads/2018/10/PREDIMED_PLUS_V1_6_Data_26_4_18_ManagPlan_and_data_sharing_Policy.pdf (accessed on 16 September 2023).
